# Autologous and micro-fragmented adipose tissue for the treatment of diffuse degenerative knee osteoarthritis

**DOI:** 10.1186/s40634-017-0108-2

**Published:** 2017-10-03

**Authors:** A. Russo, V. Condello, V. Madonna, M. Guerriero, C. Zorzi

**Affiliations:** 1 Orthopaedic Department, Sacro Cuore - Don Calabria Hospital, Via Don A. Sempreboni, 5, 37024 Negrar, VR Italy; 2 Computer Science Department, University of Verona, Verona, VR Italy

**Keywords:** Knee Chondropathy, Osteoarthritis, MSCs, Adipose tissue

## Abstract

**Background:**

Chondral lesions of the knee represent a challenge for the orthopaedic surgeon. Several treatments have been proposed with variable success rate. Recently, new therapeutic approaches, such as the use of mesenchymal stem cells, have shown promising results. The adipose tissue is a good source of these naturally occurring regenerative cells, due to its abundance and easy access. In addition, it can be used to provide cushioning and filling of structural defects. The 1-year safety and outcome of a single intra-articular injection of autologous and micro-fragmented adipose tissue in 30 patients affected by diffuse degenerative chondral lesions was evaluated.

**Methods:**

Micro-fragmented adipose tissue was obtained using a minimal manipulation technique in a closed system. The safety of the procedure was evaluated by recording type and incidence of any adverse event. The clinical outcomes were determined using the KOOS, IKDC-subjective, Tegner Lysholm Knee, and VAS pain scales taken pre-operatively and at 12 months follow-up. A level of at least 10 points of improvement in the scores has been selected as cut-off representing a clinically significant difference.

**Results:**

No relevant complications nor clinical worsening were recorded. A total median improvement of 20 points has been observed in IKDC-subjective and total KOOS, and a higher percentage of success was found in VAS pain and Tegner Lysholm Knee, where the total median improvement was 24 and 31 points, respectively.

**Conclusion:**

The results of this study show the safety and feasibility of using autologous and micro-fragmented adipose tissue in patients affected by diffuse degenerative chondral lesions. The technique is safe, minimally invasive, simple, one-step, with low percentage of complications, and compliant with the regulatory panorama.

**Electronic supplementary material:**

The online version of this article (10.1186/s40634-017-0108-2) contains supplementary material, which is available to authorized users.

## Background

Knee chondropathy represents a challenge for the orthopaedic surgeon because of the limited regenerative properties of the cartilage tissue. In the last decades, a variety of treatments, limited only to focal cartilage lesions, has been proposed (Behery et al. 2014; Seo et al. 2011; Tetteh et al. 2012; Zhang et al. 2016). For diffuse degenerative chondral lesions, no adequate biological treatments are currently available. New therapeutic approaches, such as the use of mesenchymal stem cells (MSCs) have shown promising results when applied in the context of joint degeneration (Caplan 2005; Kristjánsson and Honsawek 2014; Pak et al. 2014, 2016; Ruetze and Richter 2014). MSCs have been reported to have a perivascular origin (pericytes), and to be able to activate and influence the microenvironment by serving as “a site-regulated drug store” (Caplan and Correa 2011). Through trophic, mitogenic, anti-scarring, anti-apoptotic, immunomodulatory, and anti-microbial actions, produced by a plethora of bioactive elements, growth factors and cytokines, these cells “sense” and “signal” changes in the microenvironment where they reside (Caplan and Correa 2011). Adipose tissue is rich in vascular niches that, besides providing a readily available source of native cushioning, could serve as a smart source of MSCs (Zuk et al. 2002). The use of adipose-derived mesenchymal stem cells (ASCs) has recently created a huge interest in the context of cartilage regeneration (Pak et al. 2016; Ruetze and Richter 2014), and both in vitro and in vivo studies clearly demonstrated their anti-inflammatory and regenerative properties (Chamberlain et al. 2007). Nevertheless, enzymatic treatment and/or cell expansion have complex regulatory issues (Ährlund-Richter et al. 2009; Arcidiacono et al. 2012; Sensebé et al. 2010). Hence, the availability of a minimally manipulated autologous adipose tissue as a therapeutic option would have remarkable clinical relevance. Based on these considerations, we approached a commercially available system that intra-operatively provides micro-fragmented adipose tissue in a short time, without expansion or enzymatic treatment (Bianchi et al. 2013). With the aid of this technology, the adipose tissue is micro-fragmented and washed until free of pro-inflammatory oil and blood residues. The resulting product has been shown to be safe and effective in the treatment of different pathologies (Tremolada et al. 2016). The aim of this retrospective study is to evaluate the 1-year safety and outcome of a single intra-articular injection of autologous and micro-fragmented adipose tissue in patients affected by diffuse degenerative chondral lesions.

## Methods

### Study design and population

This is a retrospective observational study. Forty patients, affected by diffuse degenerative chondral lesions, were treated with autologous and micro-fragmented adipose tissue between 1^st^ January 2014 and 31^st^ December 2014. Out of 40 patients, 30 fulfilled the following inclusion criteria: diffuse degenerative chondral lesions of grade > II (ICRS classification), constant pain resistant to NSAIDS, and failure of conservative treatments (physiokinesitherapy, hyaluronic acid, platelet-rich plasma, corticosteroids) for at least 12 months. Patients with a history of trauma in the 6 months prior to treatment, a surgical intervention for the same indications in the previous year, synovitis, high-grade osteoarthritis (grade 4 Kellgren), axial defects >10°, systemic malignancies, metabolic disorders, and BMI > 40 were excluded from the analysis. Of these 30 patients, twenty-four (80%) had also an associated surgery (ACL/LCL reconstruction, high tibial osteotomy, meniscectomy), while six (20%) underwent arthroscopy alone. Fifteen patients (50%) underwent also previous surgeries more than one year before.

Pre-operatively and 12 months after the procedure all the patients were clinically evaluated with direct medical examination, standard X-rays and MRI. Knee Injury and Osteoarthritis Outcome Score (KOOS), International Knee Documentation Committee (IKDC)-subjective, Tegner Lysholm Knee, and Visual Analogue Scale (VAS) pain questionnaires were also collected. Based on literature data, a level of at least 10 points of improvement in the scores has been selected as a cut-off representing a clinically significant difference (Roos and Lohmander 2003; Tubach et al. 2005).

### Harvesting of the adipose tissue

The lower or the lateral abdomen was chosen as donor site for adipose tissue harvesting. Before harvesting the fat, the site was injected with carbocaine and adrenaline at very high dilution (40 ml of 2% carbocaine and 1 vial of 1 mg/ml adrenaline in 500 mL cold saline solution) by using a disposable 17G blunt cannula connected to a luer-lock 60-cm^3^ syringe. The fat was then harvested using a 13G blunt cannula, for a fast and a-traumatic suction, connected to a Vaclock® 20-ml syringe.

### Processing of the adipose tissue

**Fig. 1:**
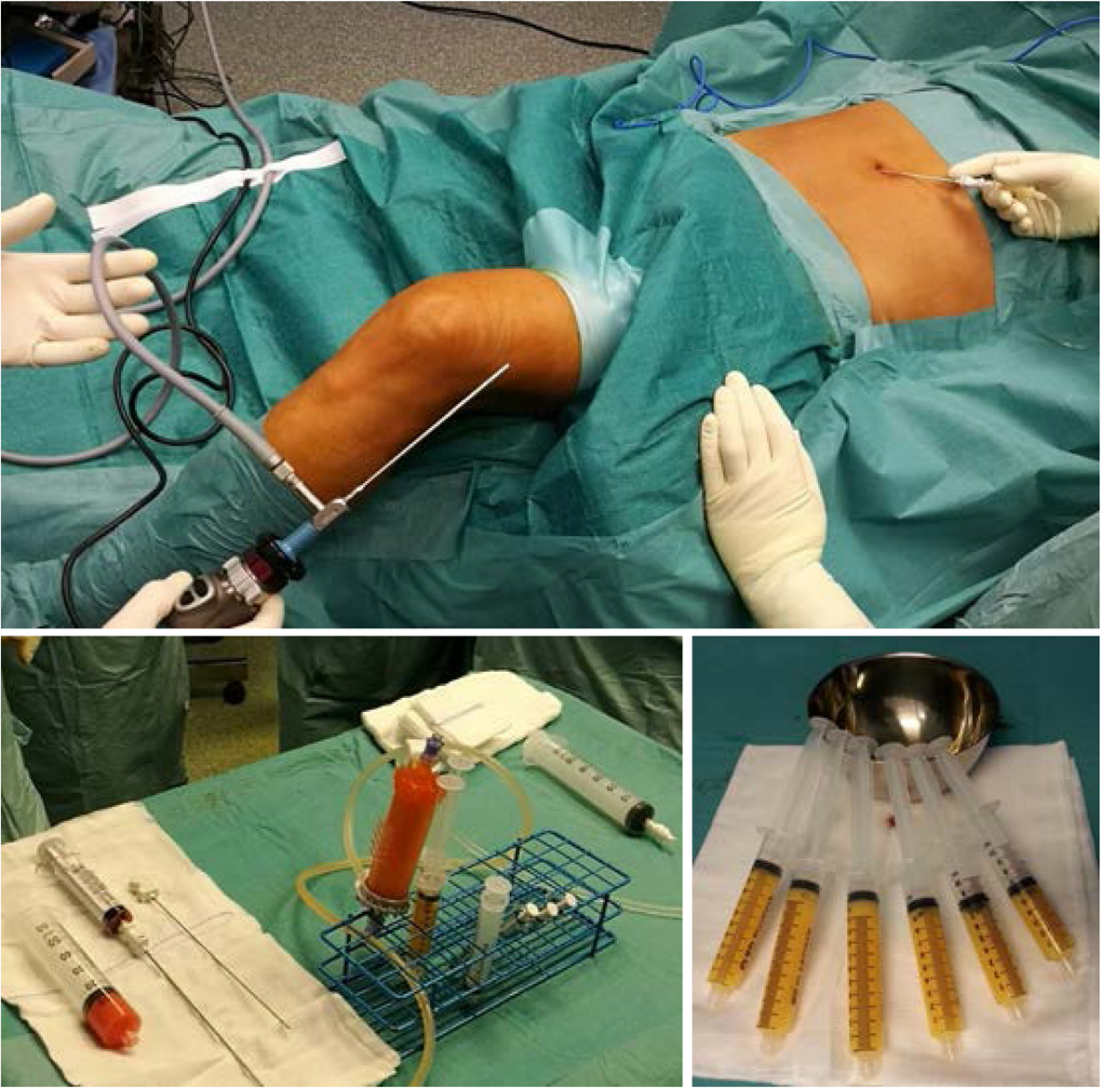
Lipogems® system. Top) surgical setting. Bottom left) device. Bottom right) final product

The harvested fat was immediately processed in the Lipogems® processing kit (Lipogems International Spa, Milan, Italy) as previously described (Bianchi et al. 2013). Lipogems® is a disposable device that progressively reduces the size of the adipose tissue clusters with a mild mechanical action while eliminating oily substances and blood residues with pro-inflammatory properties. The entire process, carried out in one surgical step, was performed in complete immersion in physiological solution minimizing any trauma to the cell products. The resulting micro-fragmented fat was collected in a 60-cm^3^ syringe, positioned for decanting the excess saline solution, and then transferred into several 10-cm^3^ syringes to be injected in the patient. Micro-fragmented fat (10–15 cm^3^) was injected intra-articular during the arthroscopic procedure (Fig. 1).

### Post-op rehabilitation protocol

All patients wore an elastic compression band on the harvesting site for 2–3 weeks. Patients were administered with painkillers in the immediate post-op upon request, and low molecular weight heparin for 15 days. The post-operative protocol was 5, 15, and 21 days of unloading for the standard arthroscopic procedures, LCA/LCL reconstructions, and high tibial osteotomies respectively. Then, full load recovery in the following 5 days. Continuous passive motion from the immediate post-op for 10 days (45 min, 4 times a day), physiokinesitherapy and proprioceptive exercises from day 5 post-op. Ice 4–5 times a day for 20 min. Suture was removed 15 days post-op.

### Safety assessment

Since the micro-fragmented fat is autologous, minimally manipulated and widely used in other surgical fields (i.e. lipofilling), safety was assessed just by evaluating local adverse events, such as fever, infections, and excessive swelling of the knee.

### Statistical analysis

Considering the relatively small sample size and the asymmetrical distribution of the data, results are expressed as median and interquartile range (IQR) for continuous variables and as percentage for categorical ones. Shapiro-Wilk test was used to test the normal distribution of continuous variables. The Wilcoxon Signed-Rank Test was employed to compare the mean of two paired groups. All the analyses were performed with STATA ver. 14 (StataCorp, 4905 Lakeway Dr., College Station, TX 77845, USA). A *p* < 0.05 was considered statistically significant.

## Results

The 30 patients (70% men and 30% women) ranged in age from 23 to 60 with a median age of 43 years old (IQR 35–52); median BMI was 26 (IRQ 24–28); 37% were smokers, 43% non-smokers, and 20% former smokers. Regarding sport activity, 7% of the patients were professionals, 47% amateurs, 23% played sport occasionally, and 23% were inactive. Eleven knees (37%) were affected by a non-focal chondral lesion of grade II, eight knees (26%) were grade III, and the remaining eleven (37%) were grade IV. Twelve patients (40%) presented a chondropathy only in one compartment, five of which in the patellofemoral, and the remaining seven in the tibiofemoral. Three patients (10%) were affected by a three-compartment chondropathy and 17 patients (57%) were affected by a patellofemoral chondropathy associated with a tibiofemoral one (Table 1). A more detailed description of the population and associated surgical procedures is reported in Additional file 1: Table S1.

**Table 1:** Demographic characteristics of the population

AGE	
p50	43
IQR	35–52
GENDER	
% M	70
% F	30
BMI	
p50	26
IQR	24–28
SMOKE	
% YES	37
% NO	43
% FORMER	20
SPORT	
% PROFESSIONALS	7
% AMATEURS	47
% OCCASIONAL	23
% NO	23
CHONDROPATHY	
% FC	73
% TP	57
% PF	70
% THREE-COMPARTMENT	30
ASS SURGERY	
% YES	80
% NO	20
PREV SURGERY	
% YES	50
% NO	50

*p50* median, *IQR* interquartile range, *FC* femoral condyle, *TP* tibial plateau, *PF* patellofemoral, *DIFF* diffuse, *ASS* associated, *PREV* previous

In general, no patients clinically worsened compared to pre-operative condition and 77% of the patients would repeat the treatment. Only three minor complications were recorded, and these required no additional treatment. In details, two cases of organized hematoma after the harvesting of the abdominal fat (one because the patient had a coagulation problem and the other because the patient was particularly slim), and one case of recurrent effusions in the first months. Noteworthy, no cases of post-operative infection, post-arthroscopic algodystrophy or stiffness were recorded.

At 12 months follow-up, an improvement of at least 10 points in the IKDC-subjective and total KOOS was observed in 70% and 67% of the patients, respectively (Table 2).

**Table 2:** Summary of the results

	Δ TEG	Δ VAS	Δ IKDC	Δ KOOS_s	Δ KOOS_p	Δ KOOS_adl	Δ KOOS_spt	Δ KOOS_QoL	Δ KOOS_tot
p50	31	−24	20	21	19	17	15	13	20
IQR	14–46	37–13	8–36	1–32	4–41	3–33	0–50	0–38	4–36
d10	87%	83%	70%	57%	63%	63%	57%	63%	67%

Data are expressed as median Δ (t_12_-t_0_). p50 = median; IQR = interquartile range; d10 = % of patients improving at least 10 points

**Fig. 2:**
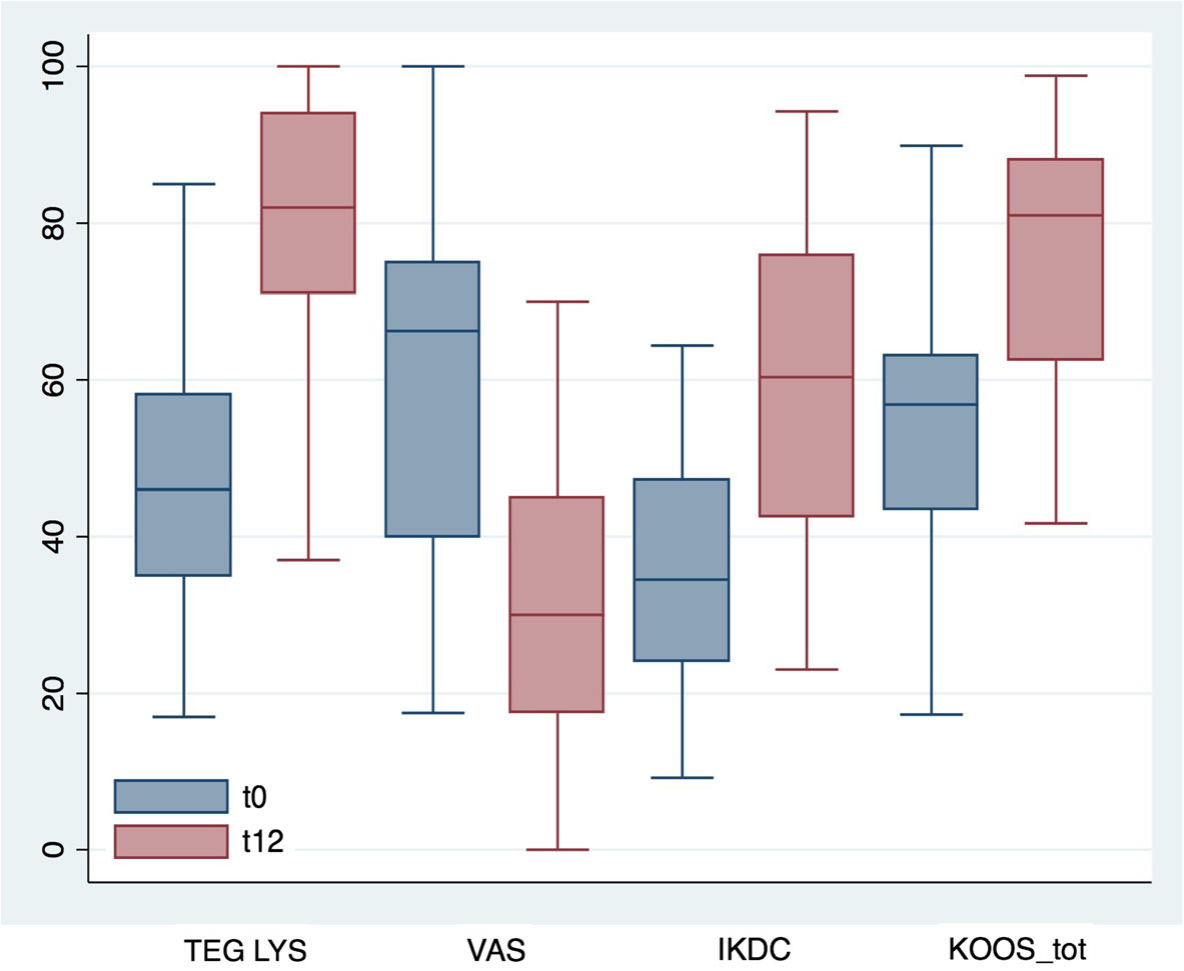
Box plot of VAS pain, Tegner Lysholm knee, IKDC subjective and total KOOS pre-operatively (t0) and at 12 months (t12) after micro-fragmented adipose tissue injection. Results are expressed as median and interquartile range

**Fig. 3:**
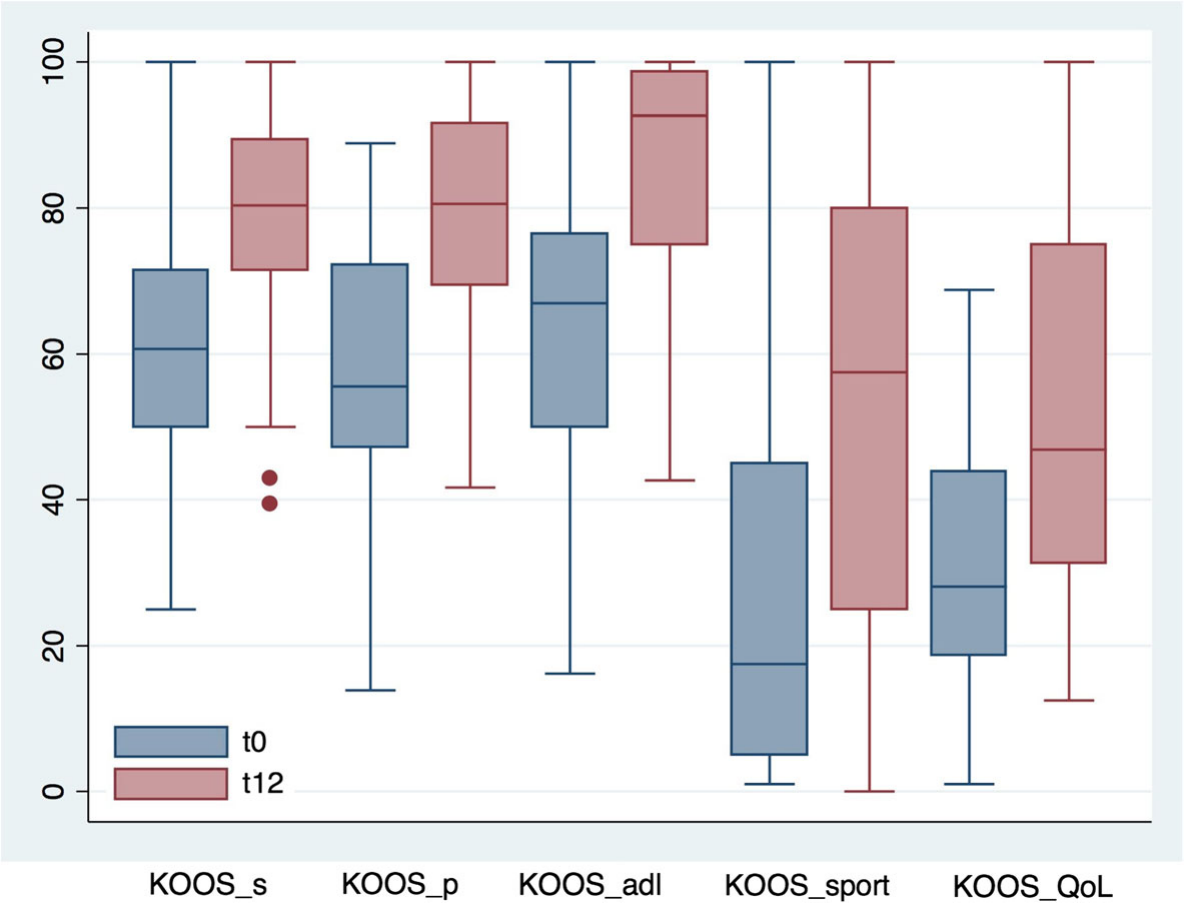
Box plot of the five KOOS subscale scores pre-operatively (t0) and at 12 months (t12) after micro-fragmented adipose tissue injection. Results are expressed as median and interquartile range. KOOS S = symptoms; KOOS P = pain; KOOS ADL = activity daily living; KOOS Spt = sport; KOOS QoL = quality of life

The total median improvement was 20 both in IKDC-subjective and in total KOOS [(IQR 8–36 and 4–36, respectively, *p* < 0.0001), Fig. 2]. Considering the five KOOS subscales, the observed median improvement was 21 (IQR 1–32) in symptoms, 19 (IQR 4–41) in pain, 17 (IQR 3–33) in activity of daily living, 15 (IQR 0–50) in sport, and 13 (IQR 0–38) in the quality of life (Fig. 3). A higher percentage of success was found in VAS pain and Tegner Lysholm Knee, where 83% and 87% of the patients, respectively, showed an improvement of at least 10 points compared to the pre-operative values, with a total median improvement of 24 (IQR 37–13, *p* < 0.0001) in VAS pain and 31 (IQR 14–46, *p* < 0.0001) in Tegner Lysholm Knee (Fig. 2 and Table 2). In general, we observed improvements of more than 20 points in more than 50% of the patients and, surprisingly, more than 50% of the patients improved of at least 30 points in VAS pain scale. Possible correlations between clinical outcomes and specific patient categories related to type and severity of chondropathy, number of affected compartments (1 or 2+), and associated or previous surgeries were also evaluated. Patients with a femoral condyle chondropathy (FC) showed higher improvements in the scores compared to patients affected by a chondropathy in any other compartment except for FC. The same trend was observed for the tibial compartment (TP). Patients affected also by a patellofemoral chondropathy (PF) improved less in the Tegner Lysholm Knee compared to patients affected by a chondropathy in any other compartment except for PF. Conversely, these patients improved more in all the other scores. Considering the number of affected compartments, patients with lesions in more than one compartment showed higher and statistically significant improvements in IKDC and in all the KOOS subscales (except for sport) compared to patients with lesions only in one compartment. With regard to the severity of chondropathy, patients affected by a chondropathy of grade I-II improved a bit more in all the scores compared to patients with grade III-IV. No significant differences in the outcomes related to previous surgeries or surgeries associated with the arthroscopy were found (Additional file 2: Table S2).

## Discussion

The main finding of this retrospective study is that autologous and micro-fragmented adipose tissue graft is a safe adjuvant for the treatment of diffuse degenerative chondral lesions. Indeed, no major complications were observed, neither at knee nor at the harvested site level and no patient worsened compared to the pre-operative condition. Although it is not possible to draw a clear conclusion about the efficacy because 80% of the patients had an associated surgery, the results of this study are very satisfactory, with the majority of the patients who significantly improved in terms of clinical outcomes with respect to baseline. Despite the lack of a control group and the association with different surgical procedures, the very positive outcome seems to point to micro-fragmented adipose tissue injection as a feasible concomitant procedure in the treatment of degenerative knee chondropathy.

We are aware that in literature a wide range of results have been reported for the same surgical procedures used alone, depending from many different parameters that can strongly affect the outcomes and their interpretation (Collins et al. 2011). Some of those results are similar to the ones we reported after the concomitant use of micro-fragmented adipose tissue graft. However, in our wide experience (more than 1500 arthroscopic procedures/year), the outcomes we obtained with the association with micro-fragmented fat injection allowed for more satisfying results if compared to the surgical procedures alone (unpublished data). The analysis of the correlations between clinical outcomes and specific patient categories was not easy to interpret because of the small number of patients that did not allow for stratification into femoral condyle or patellofemoral chondropathy. Nevertheless, it appeared that patients with lesions in more than one compartment had higher and statistically significant improvements compared to patients with lesions in only one compartment, supporting our idea of using micro-fragmented adipose tissue for the treatment of the diffuse degenerative knee pathology. The correlation with the associated surgery was also difficult to interpret, because of the heterogeneity of the sample and because only six patients had the arthroscopic procedure alone.

The injected micro-fragmented adipose tissue has been extensively studied and characterized in vitro by other authors (Bianchi et al. 2013; Ceserani et al. 2016; García-Contreras et al. 2015). The findings show that it contains an abundant number of cells able to secrete a variety of bioactive molecules that act through a paracrine mechanism to prime and sustain angiogenic, anti-fibrotic, anti-apoptotic, anti-microbial and immunomodulatory responses in the target tissue. Compared to ASCs, micro-fragmented adipose tissue does not require any enzymatic treatment and it potentially overcomes the large number of processing steps, the high economic burden, and the complex restrictions related to cell expansion and extensive manipulation (Ährlund-Richter et al. 2009; Arcidiacono et al. 2012; Sensebé et al. 2010). The procedure is simple and can be used “last second” in the OR without previous preparation, and, in case of failure, additional future treatments are not precluded. It is sustainable, quick, one-step, minimally invasive, and with very low percentage of complications (no cases of infection, fibrosis, post-arthroscopic femoral condyle necrosis or algodystrophy). In addition, it complies with the regulatory panorama because the adipose tissue is autologous, minimally manipulated, and intended for homologous use (Lysaght and Campbell 2011). Indeed, it is well known that the joint cavity of the knee has a large adipose cushion, the Hoffa’s pad, which, besides a biomechanical function, plays a fundamental role in ensuring homeostasis of the cartilage and of the extracellular matrix of the articular space (Ioan-Facsinay and Kloppenburg 2013). In this context, micro-fragmented adipose tissue could act as a natural “bioreactor” providing cushioning and filling and stimulating the natural regenerative properties of the tissue itself.

The main limitation of the study is the heterogeneity of the population and the associated surgical procedures. Indeed, in quality of early users of this innovative treatment, because of the initial lack of specific indications, we made use of this approach in different conditions and in association with various surgical procedures.

Despite these confounding factors, the study allowed to collect very positive initial data to ascertain the safety of the technique posing important basis for the future randomized controlled study.

## Conclusion

In conclusion, our results point to autologous and micro-fragmented adipose tissue injection as an innovative and safe approach for the treatment of diffuse degenerative knee chondropathy. The procedure is simple, economic, quick, minimally invasive, one-step, with low percentage of complications, and compliant with the regulatory panorama. Despite the small number of patients and the heterogeneity of the population, the results are very positive and promising, and encourage the initiation of future randomized and controlled studies.

## Additional files

Additional file 1: Table S1. Descriptive features of the population. (DOCX 22 kb)

Additional file 2: Table S2. Correlations between clinical outcomes and specific patient categories. (PDF 72 kb)

## Abbreviations

ACL: Anterior cruciate ligament
ASCs: Adipose-derived stem cells
FC: Femoral condyle
IKDC: International knee documentation committee
IRQ: Interquartile range
KOOS: Knee injury and osteoarthritis outcome score
LCL: Lateral collateral ligament
MSCs: Mesenchymal stem cells
NSAIDs: Nonsteroidal anti-inflammatory drugs
PF: Patellofemoral
SVF: Stromal vascular fraction
TP: Tibial plateau
VAS: Visual analogue scale
