# Supplementary material for: Autologous and micro-fragmented adipose tissue for the treatment of diffuse degenerative knee osteoarthritis
Source: J Exp Orthop. 2017 Oct 3;4:33. doi: 10.1186/s40634-017-0108-2 (PMC5626678; doi:10.1186/s40634-017-0108-2)
Supplement: Supplementary file 1 — Descriptive features of the population. (DOCX 22 kb) [file 40634_2017_108_MOESM1_ESM.docx]

| **Additional file 1: Table S1.** Descriptive features of the population | | | | | | | | | | | | | | | | | | | | | | | | | | | |
| --- | --- | --- | --- | --- | --- | --- | --- | --- | --- | --- | --- | --- | --- | --- | --- | --- | --- | --- | --- | --- | --- | --- | --- | --- | --- | --- | --- |
| **ID** | **GRADE OF CHONDROPATHY** | | | | **SURGERY** | **ASS. SURGERY** | **DESCRIPTION** | | | **TEG LYS** | | **VAS** | | **IKDC** | | **KOOS S** | | **KOOS P** | | **KOOS ADL** | | **KOOD SPT** | | **KOOS QoL** | | **KOOS TOT** | |
|  | **FC** | **TP** | **PF** | **DIFF** |  |  |  |  |  | **T0** | **T12** | **T0** | **T12** | **T0** | **T12** | **T0** | **T12** | **T0** | **T12** | **T0** | **T12** | **T0** | **T12** | **T0** | **T12** | **T0** | **T12** |
| **1** | III M | II |  |  | ARTH | YES | Medial meniscal scaffold implant | | | 40 | 78 | 65 | 55 | 40 | 51 | 100% | 96% | 78% | 92% | 91% | 94% | 45% | 45% | 19% | 19% | 76 | 81 |
| **2** | III | III | III | III | ARTH | YES | Lateral meniscal regularization | | | 79 | 100 | 68 | 38 | 48 | 93 | 68% | 100% | 56% | 97% | 71% | 100% | 50% | 80% | 44% | 100% | 63 | 97 |
| **3** | IV L | IV L | II |  | ARTH | YES | Medial collateral cyst removal | | | 17 | 50 | 83 | 38 | 24 | 40 | 29% | 71% | 22% | 81% | 38% | 82% | 5% | 25% | 6% | 25% | 27 | 68 |
| **4** | II M |  |  |  | ARTH | YES | Medial meniscal scaffold implant | | | 58 | 71 | 48 | 23 | 47 | 70 | 79% | 79% | 78% | 86% | 76% | 93% | 55% | 80% | 38% | 44% | 71 | 83 |
| **5** |  |  | III |  | ARTH | YES | HOFFA’s body fibrosis removal | | | 46 | 37 | 35 | 35 | 35 | 32 | 79% | 79% | 75% | 75% | 79% | 78% | 5% | 5% | 13% | 13% | 63 | 63 |
| **6** | II M | II M | II |  | ARTH | YES | Notchplasty + cyclops removal + plate and screw removal | | | 43 | 54 | 75 | 60 | 15 | 23 | 61% | 68% | 50% | 42% | 43% | 43% | 0% | 0% | 0% | 44% | 38 | 42 |
| **7** |  | IV |  |  | ARTH | YES | Chondral shaving, ACL and LCL reconstruction | | | 21 | 79 | 80 | 45 | 35 | 53 | 29% | 39% | 56% | 72% | 57% | 74% | 0% | 25% | 38% | 75% | 44 | 62 |
| **8** | II M | II M |  |  | ARTH | YES | Medial selective meniscectomy + parameniscal cyst removal | | | 32 | 100 | 80 | 8 | 28 | 89 | 71% | 100% | 50% | 92% | 50% | 99% | 25% | 95% | 38% | 88% | 47 | 96 |
| **9** | III M |  |  |  | ARTH | YES | Plate and TIB screw removal | | | 55 | 94 | 75 | 70 | 43 | 43 | 86% | 86% | 53% | 56% | 68% | 69% | 35% | 35% | 43% | 44% | 61 | 63 |
| **10** | II E | II L | II E |  | ARTH | YES | External meniscus regularization + ACL reconstruction | | | 46 | 100 | 68 | 0 | 30 | 84 | 54% | 100% | 50% | 97% | 66% | 100% | 10% | 70% | 50% | 75% | 52 | 94 |
| **11** | IV M, II L | IV M, II L | III |  | ARTH | YES | Osteotomy +medial meniscus regularization | | | 48 | 79 | 45 | 25 | 35 | 60 | 61% | 89% | 67% | 83% | 75% | 93% | 35% | 45% | 25% | 44% | 61 | 80 |
| **12** | IV M | III M | III |  | ARTH | YES | Medial selective meniscectomy | | | 24 | 100 | 100 | 70 | 9 | 86 | 38% | 86% | 14% | 81% | 16% | 100% | 0% | 95% | 19% | 75% | 17 | 91 |
| **13** | II L |  |  |  | ARTH | YES | Distal femoral osteotomy | | | 65 | 90 | 40 | 30 | 47 | 77 | 68% | 71% | 78% | 92% | 90% | 99% | 15% | 70% | 63% | 75% | 72 | 87 |
| **14** | IV M |  |  |  | ARTH | YES | High Tibial osteotomy | | | 46 | 74 | 70 | 55 | 26 | 35 | 68% | 71% | 47% | 56% | 65% | 69% | 5% | 5% | 13% | 13% | 49 | 54 |
| **15** | II M |  | II |  | ARTH | YES | Anterior tibial tuberosity screw removal | | | 44 | 85 | 68 | 13 | 20 | 75 | 50% | 86% | 14% | 81% | 35% | 94% | 5% | 55% | 0% | 50% | 26 | 81 |
| **16** | IV M, II L | IV M, II L | III |  | ARTH | YES | Plate removal | | | 76 | 78 | 18 | 45 | 63 | 67 | 93% | 89% | 83% | 69% | 91% | 81% | 25% | 25% | 44% | 44% | 77 | 70 |
| **17** | IV M | IV M | III |  | ARTH | YES | Medial and lateral meniscus regularization | | | 66 | 84 | 63 | 18 | 24 | 62 | 61% | 96% | 56% | 81% | 68% | 81% | 100% | 100% | 25% | 31% | 64 | 81 |
| **18** |  | III L | IV |  | ARTH | YES | Patellar and trochlear osteophytes removal | | | 46 | 94 | 18 | 18 | 35 | 85 | 43% | 89% | 89% | 86% | 65% | 97% | 0% | 75% | 31% | 69% | 55 | 88 |
| **19** |  |  | II |  | ARTH | YES | Patellar tendon scarification and patellar bone drilling | | | 72 | 89 | 40 | 28 | 45 | 59 | 68% | 89% | 69% | 92% | 76% | 94% | 20% | 55% | 25% | 38% | 62 | 83 |
| **20** |  |  | III |  | ARTH | YES | Medial and lateral cyst removal | | | 80 | 80 | 30 | 25 | 53 | 48 | 88% | 79% | 78% | 81% | 100% | 100% | 100% | 100% | 69% | 69% | 90 | 89 |
| **21** | IV M | III | III | III | ARTH | YES | Medial and lateral selective meniscectomy | | | 46 | 50 | 50 | 30 | 26 | 36 | 54% | 61% | 58% | 58% | 62% | 66% | 5% | 10% | 50% | 50% | 52 | 55 |
| **22** | III M |  |  |  | ARTH | YES | Medial meniscus regularization | | | 49 | 94 | 70 | 5 | 53 | 75 | 57% | 82% | 50% | 97% | 79% | 91% | 0% | 75% | 25% | 100% | 55 | 90 |
| **23** | IV M | IV M | II |  | ARTH | YES | Plate and screw removal + fibrosis debridement | | | 54 | 84 | 33 | 13 | 54 | 71 | 50% | 71% | 47% | 83% | 56% | 99% | 15% | 65% | 25% | 50% | 45 | 82 |
| **24** |  |  | II |  | ARTH | YES | Synovial plica removal + patellar tendon cruentation and drilling | | | 85 | 95 | 78 | 53 | 64 | 76 | 71% | 79% | 72% | 81% | 100% | 100% | 70% | 75% | 63% | 63% | 82 | 86 |
| **25** |  |  | III |  | ARTH | NO | n.a. | | | 26 | 85 | 88 | 30 | 15 | 44 | 38% | 71% | 31% | 75% | 37% | 87% | 5% | 35% | 7% | 19% | 29 | 69 |
| **26** | III M |  | II |  | ARTH | NO | n.a | | | 58 | 70 | 38 | 0 | 30 | 94 | 71% | 93% | 64% | 100% | 66% | 100% | 20% | 100% | 38% | 100% | 58 | 99 |
| **27** |  | II |  |  | ARTH | NO | n.a. | | | 50 | 71 | 70 | 50 | 38 | 44 | 82% | 79% | 61% | 53% | 72% | 75% | 25% | 25% | 19% | 13% | 61 | 59 |
| **28** | II | II | II | II | ARTH | NO | n.a. | | | 26 | 72 | 38 | 15 | 17 | 41 | 25% | 50% | 28% | 61% | 43% | 71% | 100% | 100% | 31% | 44% | 42 | 66 |
| **29** | II M |  | IV |  | ARTH | NO | n.a. | | | 34 | 65 | 73 | 45 | 23 | 30 | 43% | 43% | 19% | 58% | 31% | 60% | 0% | 0% | 13% | 25% | 25 | 46 |
| **30** | I M | I M | II |  | ARTH | NO | n.a. | | | 35 | 85 | 65 | 30 | 38 | 61 | 54% | 86% | 53% | 81% | 72% | 94% | 50% | 60% | 44% | 75% | 60 | 84 |
| FC= femoral condyle; TP= tibial plateau; PF= patellofemoral; DIFF=diffuse; M=medial; L=lateral; E=external; ARTH = arthroscopy;  TEG LYS=Tegner Lysholm knee; VAS=visual analogue scale; KOOS S=symptoms; KOOS P=pain; KOOS ADL=activity daily living; KOOS SPT=sport; KOOS QoL=quality of life; KOOS TOT=total. | | | | | | | | | | | | | | | | | | | | | | | | | | | |
