# Supplementary material for: Autologous and micro-fragmented adipose tissue for the treatment of diffuse degenerative knee osteoarthritis
Source: J Exp Orthop. 2017 Oct 3;4:33. doi: 10.1186/s40634-017-0108-2 (PMC5626678; doi:10.1186/s40634-017-0108-2)
Supplement: Supplementary file 2 — Correlations between clinical outcomes and specific patient categories. (PDF 72 kb) [file 40634_2017_108_MOESM2_ESM.pdf]

Additional file 2: Table S2 Correlations between clinical outcomes and specific patient categories

	Obs	TEG	VAS	IKDC	KOOS_s	KOOS_p	KOOS_adl	KOOS_sport	KOOS_QoL	KOOS_tot
**FC**										
NO	8	32.80556	-29.91071	24.33036	20.78776	7.561116	13.59352	91.07143	25	18.99374
YES	22	67.74621	-48.53801	62.08398	41.4286	54.57629	27.07336	52.72727	73.21429	47.36633
P value		*0.4530*	*0.0746*	*0.0913*	*0.3850*	*0.0785*	*0.2225*	*0.7392*	*0.1782*	*0.1594*
**TP**										
NO	13	38.46154	-32.25806	30.80357	5.260831	17.85806	14.82181	45.45454	20	20.69444
YES	17	95	-44.44444	66.80498	47.31892	44.9964	30.61338	60	75	42.29885
P value		*0.0687*	*0.7855*	*0.1373*	*0.0514*	*0.9166*	*0.3792*	*0.8485*	*0.6129*	*0.6009*
**PF**										
NO	9	70.90909	-28.57143	41.20983	5.260831	17.85806	9.83168	45.45454	16.66667	16.80791
YES	21	55.55556	-44.44444	66.80498	47.31892	52.63357	41.66312	60	75	53.72425
P value		*0.3535*	*0.5411*	*0.2133*	*0.0249*	*0.2977*	*0.1816*	*0.9634*	*0.1787*	*0.1819*
**COMPART.**										
1	12	40.23077	-29.91071	30.74269	5.260831	14.60503	8.323351	26.2987	8.678557	12.65497
2+	18	92.17914	-49.1453	83.04348	47.34057	65.75051	50.54766	80	100	57.64502
P value		*0.2041*	*0.1440*	*0.0067*	*0.0148*	*0.0943*	*0.0177*	*0.8300*	*0.0348*	*0.0223*
**FC**										
I-II	9	93.18182	-53.84615	63.48195	39.9972	83.34	51.10305	45.45454	71.42857	56.26477
III-IV	13	60.86956	-44.44444	41.20983	42.86	44.9964	19.55654	60	75	30.17945
P value		*0.2705*	*0.3851*	*0.2426*	*0.7891*	*0.4225*	*0.2852*	*0.6855*	*0.4210*	*0.3006*
**TP**										
I-II	7	117.3913	-53.84615	60.68602	39.9972	52.63357	30.61338	0	50	41.0084
III-IV	10	60.06945	-44.09722	70.06916	47.34057	37.491	34.93793	216.6667	100	48.01155
P value		*0.5582*	*0.5579*	*1.0000*	*0.3798*	*1.0000*	*0.6256*	*0.0753*	*0.4929*	*0.5582*
**PF**										
I-II	10	74.36869	-57.27273	63.7455	51.42813	66.49542	51.10305	254.1667	85.71429	62.86652
III-IV	11	27.27273	-37.93103	73.33334	47.31892	24.98875	23.53333	28.57143	75	30.17945
P value		*0.4386*	*0.0484*	*0.6221*	*0.3979*	*0.2748*	*0.3973*	*0.5703*	*0.2872*	*0.2908*
**ASS SURG**										
NO	6	117.0168	-56.92308	100.6918	44.99883	88.24871	58.30651	10	85.71429	62.86652
YES	24	58.21256	-36.12903	51.0828	34.1203	21.42341	20.35758	80	50	28.669
P value		*0.2762*	*0.1693*	*0.5338*	*0.7753*	*0.1947*	*0.1136*	*0.3575*	*0.7940*	*0.3507*
**PREV SURG**										
NO	15	60.86956	-43.75	60.68602	36.66087	44.9964	28.21273	60	75	41.0084
YES	15	70.90909	-28.57143	48.83227	11.7773	17.85806	14.82181	45.45454	40	20.69444
P value		*0.9504*	*0.4066*	*0.5476*	*0.5198*	*0.2715*	*0.3612*	*0.9664*	*0.7382*	*0.4429*

Data are expressed as median Δ $[(t_{12}-t_0)/t_0]*100$. FC= femoral condyle; TP= tibial plateau; PF= patellofemoral; COMPART=affected compartments; ASS SURG= associated surgery; PREV SURG=previous surgery. TEG=tegner lysholm knee; VAS=visual analogue scale; KOOS s=symptoms; KOOS p=pain; KOOS adl=activity daily living; KOOS spt=sport; KOOS QoL=quality of life; KOOS tot=total. P<0.05 was considered statistically significant.
